# The evidence base for chiropractic treatment of musculoskeletal conditions in children and adolescents: The emperor's new suit?

**DOI:** 10.1186/1746-1340-18-15

**Published:** 2010-06-02

**Authors:** Lise Hestbaek, Mette Jensen Stochkendahl

**Affiliations:** 1 Nordic Institute of Chiropractic and Clinical Biomechanics, Forskerparken 10, DK-5230 Odense M, Denmark

## Abstract

Five to ten percent of chiropractic patients are children and adolescents. Most of these consult because of spinal pain, or other musculoskeletal complaints. These musculoskeletal disorders in early life not only affect the quality of children's lives, but also seem to have an impact on adult musculoskeletal health. Thus, this is an important part of the chiropractors' scope of practice, and the objective of this review is to assess the evidence base for manual treatment of musculoskeletal disorders in children and adolescents.

Randomized, quasi-randomized and non-randomized clinical studies were included if they investigated the effect of manual therapy on musculoskeletal disorders in children and/or adolescents. The MEDLINE and MANTIS databases were searched, and studies published in English, Danish, Swedish or Norwegian were included.

Only three studies were identified that in some way attempted to look at the effectiveness of manual therapy for children or adolescents with spinal problems, and none of these was a randomized controlled clinical trial. As for the rest of the musculoskeletal system, only one study of temporomandibular disorder was identified.

With this review, we have detected a paradox within the chiropractic profession: Although the major reason for pediatric patients to attend a chiropractor is spinal pain, no adequate studies have been performed in this area. It is time for the chiropractic profession to take responsibility and systematically investigate the efficiency of joint manipulation of problems relating to the developing musculoskeletal system.

## Background

All over the world, chiropractors treat a large variety of conditions in many ways. However, the core area of chiropractic practice is the musculoskeletal system, with special focus on the spine. Surveys of chiropractic patients in different countries have shown that spinal pain is the most common reason for seeking chiropractic care with 64%-86% reporting spine-related symptoms [1-5]. Other disorders related to the musculoskeletal system are also quite common, whereas non-musculoskeletal problems represent only 2-6% of the complaints [1-5]. Most of the patients in these surveys were adults, but chiropractors also treat children all over the world although the proportion of pediatric patients may vary between countries. In a Danish survey, 7% of the patients were under the age of 20 [6], in a Swedish survey it was well below 5% [3], whereas 11% of the patients were children and adolescents in a survey from Boston, Massachusetts, United States (US) [7].

There are only few descriptions of pediatric patients' use of chiropractic services. A Danish survey showed that 64% of patients, aged 2 to 18, had primary complaints from the musculoskeletal system and 13% had headache as primary complaint [8]. A report from the National Center for Health Statistics demonstrated that 12% of US children used some type of complimentary or alternative medicine (CAM), with manipulation being the most common. The most frequent complaint causing children to seek CAM care, in general, was back and neck pain [9]. Overall, there might be larger variety of symptoms among children and adolescents in chiropractic practice than among adult patients, but musculoskeletal disorders are by far the most prevalent complaints.

These disorders deserve more focus than they receive at present. Traditionally, spinal pain has been considered an ailment of adulthood. However, there is a growing understanding that such problems originate early in life with up to 50% of children and adolescents experiencing low back pain within the course of a year and a third of these will experience recurrent pain [10-12]. Even more importantly, low back pain in adolescence seems to track into adult life and is a predictor for later low back pain [13,14]. Also neck pain and headache are quite prevalent in young populations [15-18] and seem to be carried forward into adulthood [18,19].

Chiropractic treatment covers a range of non-surgical and non-medical types of treatment such as exercise, dietary advice, ergonomic advice, soft tissue treatment and others, but the core treatment for the chiropractic profession is joint manipulation[20,21]. The evidence base for treating musculoskeletal disorders with manipulation has been steadily growing over the past two decades so there is now substantial evidence supporting this type of treatment [22-24] and spinal manipulation for back pain is recommended in several guidelines[25,26]. The research forming this evidence base relates to the adult population and the guidelines only concern adult back pain. There are no indications in the literature that children would or would not respond to treatment in the same manner as adults. Logically, there might be considerations for treating children with manual therapy that do not apply to adults [27] and therefore, treating children with therapies only tested in adult populations is uncertain ground for any type of treatment, including manipulation, and research should be carried out on this age group specifically.

Since musculoskeletal disorders are the most common disorders seen in pediatric patients in chiropractic practice, we decided to do a systematic literature review of the effectiveness of manual therapy for musculoskeletal disorders in children and adolescents. However, as it turned out, there was hardly anything to review, turning this into a very brief report.

## The review

### Type of studies

Randomized, quasi-randomized (allocation subject to bias) and non-randomized clinical studies were included if they investigated the effect of manual therapy on musculoskeletal disorders in children and/or adolescents. Therefore, we excluded reviews, case reports/series, letters, editorials, guidelines and comments.

### Type of participants

Children and adolescents (2-18 years of age) with musculoskeletal disorders. Studies relating to infants below the age of two were excluded due to uncertain diagnoses. Studies of fractures, dislocations or structural anomalies were excluded.

### Type of intervention

All types of manual therapy.

### Limitations

Only studies published in the peer-reviewed literature in English, Danish, Swedish or Norwegian were included.

### Search methods for identification of studies

MEDLINE and MANTIS were searched from their respective beginning to December 2009. In MEDLINE, the following search strategy was used: ("Manipulation, Spinal" [Mesh], "Manipulation, Osteopathic" [Mesh], "Manipulation, Chiropractic" [Mesh], "Chiropractic" [Mesh] and "Manipulation, Orthopedic" [Mesh]) NOT case report NOT fracture. A similar strategy was used in MANTIS in an adapted form. Finally, the reference lists of relevant reviews were screened.

### Selection of studies

Both authors independently screened the titles and abstracts from the search results. Potentially relevant papers were obtained in full text and independently assessed for inclusion.

### Qualitative and quantitative analyses

Data were not extracted nor pooled for meta-analysis because the included studies were few and disparate and data could not rationally be pooled. For the same reasons, no attempt at evaluating the methodological quality was done. The identified studies are simply briefly described.

### Results

The MEDLINE search revealed 478 titles, of which three articles were retained in full text [28-30]. The search in MANTIS resulted in one additional article [31]. Three of the four articles related to spinal disorders: **1**) a cohort time-series trial investigating the effect of chiropractic interventions on small scoliotic curves. This study concluded that chiropractic was not effective in reducing the severity of scoliotic curves [28], **2**) a prospective cohort study evaluating chiropractic management of pediatric patients with low back pain, including 54 patients. This study concluded that patients responded favorably to chiropractic management with no reported complications [29]. In neither of these two studies could the results for the treated group of patients be compared to natural history or other types of treatment and **3**) a pilot study published in 2006, investigating chiropractic manipulation in adolescent idiopathic scoliosis including six patients [31]. The conclusion of the pilot study was that a large scale study was feasible, but to our knowledge, no report of such a large scale study is available and we were unable to find such a trial registered at http://controlled-trials.com or at http://clinicaltrials.gov, indicating the larger study is not being performed. The fourth, and the only non-spinal, study pertained to the temporomandibular joint. This was a randomized controlled trial of 28 children evaluating osteopathic manipulative treatment (OMT) of temporomandibular disorders. The results suggest that OMT can improve mandibular kinematics [30].

### Post-hoc search

In light of the very few studies that met our inclusion- and exclusion criteria, we wanted to obtain a superficial overview of all the published literature in the field. We used the same search terms in MEDLINE, but limited the search to different types of publications. There were 32 reviews, 118 letters, editorials, addresses or comments, and 113 case reports/case series. We screened the titles of the case reports and found 35 studies of dislocations. Excluding dislocations, we were able to identity 23 studies describing the effect of manual therapy in children. Eleven of these dealt with musculoskeletal disorders and 12 with non-muscular disorders. The objective of the rest of the case studies could not be determined based on the titles alone.

## Discussion

Only three studies were identified that in some way attempted to look at the effectiveness of joint manipulation for children or adolescents with spinal problems, and none of these was a randomized controlled clinical trial. In other words, there is no first level evidence available in relation to the effectiveness of manual therapy for spinal disorders in the young population. As for the rest of the musculoskeletal system, only one randomised trial of temporomandibular disorder was identified.

We might have missed some studies due to a limited search strategy. Mainly, we only searched the Medline and MANTIS databases and might have missed articles not indexed there. We did not have access to Embase, which could have expanded the search. There are probably also reports in the non-indexed literature, the so-called "grey literature", which we have not included. We intentionally did not search the grey literature, since there is no minimum of quality control in such publications and they are not accessible to the majority of health care providers. However, we believe that we have not missed a body of literature large enough to change the overall impression of a poorly researched area.

With this review, we have detected a paradox within the chiropractic profession: Although the major reason for children and adolescents [8] as well as adults [2-4], to attend a chiropractor is spinal pain, no adequate studies have been performed in this area.

If the chiropractic profession wishes to undertake the task of caring for children's musculoskeletal problems, an important area for which no other profession has taken responsibility, we can not simply treat children as small adults. We must build up scientifically sound knowledge focusing on the developing spine. We should also provide the opportunity to specialize in pediatrics. To do this, we are in dire need of evidence on which to build daily practice as well as a specialist education. It is necessary to produce research that documents the type of pediatric patients chiropractors treat, the type of treatments offered, the effect of these treatments, and potential side effects. This means that randomized controlled clinical trials evaluating the most common treatments for the most common conditions in chiropractic pediatric patients must be performed, including proper registration of adverse effects.

It is time for the chiropractic profession to take responsibility and make an effort to provide an evidence-based treatment for problems relating to the developing musculoskeletal system.

## Conclusion

That which appeared at first to be a large body of literature in relation to chiropractic treatment of children turned out to be a case of the emperor's new suit. Of the almost 500 identified titles, there were several hundred case studies, reviews, letters etc., but only four clinical studies related to the *effect *of manual therapy on musculoskeletal problems in children, one of which was a pilot study and two lacked a control group. It is long overdue that we, as caretakers of musculoskeletal health, face reality. As the story goes: "*But he has nothing on at all," said a little child at last. ...... "But he has nothing on at all," cried at last the whole people. That made a deep impression upon the emperor, for it seemed to him that they were right; but he thought to himself, "Now I must bear up to the end." And the chamberlains walked with still greater dignity, as if they carried the train which did not exist*." [32]. However, we as a profession must not "bear it up to the end". Therefore, we must stop making reviews and recommendations based upon hot air and instead start creating a proper robe for the emperor. He needs it.

## Competing interests

As researchers, our interest is to further research in this area. This might be considered a competing interest by some. The views expressed in this manuscript are our sincere opinion to promote evidence-based treatment of children for the common good.

## Authors' contributions

Both authors independently performed the literature search and the screening of titles and abstracts. LH drafted the manuscript, MJS did the critical revising and both authors read and approved the final manuscript.
